# Mitigating Silica Fouling and Improving PPCP Removal by Modified NF90 Using In Situ Radical Graft Polymerization

**DOI:** 10.3390/membranes11110904

**Published:** 2021-11-22

**Authors:** Yi-Li Lin, Nai-Yun Zheng, Hao-Yu Gan, An-Xian Chang, Huai-Xuan Luo, Yao-Jie Mao

**Affiliations:** Department of Safety, Health and Environmental Engineering, National Kaohsiung University of Science and Technology, Kaohsiung 824, Taiwan; naiyun@nkust.edu.tw (N.-Y.Z.); u0313054@nkust.edu.tw (H.-Y.G.); afsd124afsd@gmail.com (A.-X.C.); u0313074@nkust.edu.tw (H.-X.L.); u0313006@nkust.edu.tw (Y.-J.M.)

**Keywords:** in situ membrane modification, nanofiltration, fouling mitigation, pharmaceutical and personal care products (PPCPs), fouling mechanism

## Abstract

This study in-situ modified a commercial nanofiltration membrane, NF90, through the concentration-polymerization-enhanced radical graft polarization method by applying two agents of 3-sulfopropyl methacrylate potassium salt (SPM) and 2-hydroxyethyl methacrylate (HEMA) with different dosages. Surface characterization revealed that the modified membranes became rougher and more hydrophilic compared with the pristine membrane. The modified membranes exhibited considerably enhanced separation performance with 5.8–19.6% higher NaCl rejection and 17.2–19.9% higher pharmaceuticals and personal care products (PPCPs) rejection than the pristine membrane. When treating the feedwater with high silica concentration, the modified membranes exhibited relatively less flux decline with high percentage of reversible fouling, especially the ones modified using a lower monomer concentration (0.01 M SPM and 0.01 M HEMA). Moreover, membrane modification enhanced the PPCP rejection (1.3–5.4%) after silica fouling by mitigating foulant deposition on the membrane surface. The fouling mechanism was confirmed to be intermediate blocking of membrane pores. Therefore, the in-situ modification technique with a low monomer concentration proved to be effective for mitigating silica fouling and improving PPCP rejection, which can be easily performed and cost-effective in practical application.

## 1. Introduction

Among various water treatment technologies, nanofiltration (NF) and reverse osmosis (RO) membrane separation have been widely applied for rejecting emerging contaminants, such as endocrine-disrupting compounds (EDCs) and pharmaceuticals (PhACs)/ pharmaceuticals and personal care products (PPCPs) [1], producing fresh water for drinking water supplies [2], wastewater reclamation [3], and desalination [4,5]. However, membrane fouling has always remained as the major challenge for the application of NF and RO. The fouling types can be classified as inorganic scaling (the deposition of hardness scales or minerals such as CaCO_3_, CaSO_4_, and Ca_3_(PO_4_)_2_) [6], organic scaling (such as natural organic matter (NOM), humic acid, and the derivatives of humic substances) [7,8], colloidal fouling (such as suspended particles) [9], and biofouling (such as polysaccharides, protein, and extracellular polymeric substances (EPS)) [10]. Membrane fouling can lead to reducing permeability productivity, deteriorating permeate quality, increasing energy consumption and treatment cost through the addition of chemical cleaning agents or the scale inhibitors [11], and decreasing membrane life span [10]. To overcome the abovementioned issues, it is essential to develop or modify the membranes to achieve high surface hydrophilicity and selectivity for increasing solute rejection and mitigating fouling [4,12]. Compared to develop a new membrane from the beginning, it can be more efficient and cost-effective to modify the commercial membranes.

There are various membrane modifying technologies that have been reported, such as surface grafting [12,13], surface coating [14], and incorporation of nanoparticles as nano-filters on the surface of composite membranes [15]. In our previous study, we conducted in-situ radical graft polymerization to modify a widely used commercial NF membrane (NF270) using low bulk concentrations of monomers (3-sulfopropyl methacrylate potassium salt (SPM) and 2-hydroxyethyl methacrylate (HEMA)) and initiators that could be increased on the membrane surface thanks to the inevitable concentration polarization of the rejected solutes on a dense membrane [16,17]. Moreover, using low bulk concentrations of monomers and initiators is economical and beneficial for avoiding the environmental burden caused by the discharged of residual concentration. SPM with strong negatively charged sulfonic groups could enhance the electrostatic repulsion effect to pollutants and simultaneously mitigate fouling [13,18]. On the other hand, HEMA with many hydroxyl and hydrogen groups could improve the membrane surface hydrophilicity and enhance the rejection mechanism of steric hindrance [13]. Relevant studies have shown that the modified membranes using in-situ radical graft polymerization could remarkably improve fouling reversibility and increase salt and contaminant removal efficiency [13,16,19]. Therefore, we adopted the technology of the concentration-polymerization-enhanced radical graft polarization method to modify a common commercial membrane (NF90). This technology is cost-effective, easy to operate with reproducibility, and can enhance separation performance of trace contaminant by the modified membranes.

Recently, major attention has been paid to the emerging contaminants, namely PPCPs [17,20,21], which are commonly found and frequently detected in aqueous environment, including drinking water sources, sewage treatment plants (STPs), and waste water treatment plants (WWTPs) at trace-level ng L^−1^ to μg L^−1^ [3,20]. These microcontaminants may have potentially adverse health and ecological impacts, but cannot be effectively removed by conventional water treatment processes. Currently, NF and RO membranes are often considered effective for PPCP rejection in the tertiary treatment process [20,21]. For example, Khanzada et al. used NF membrane coated using silver nanoparticles (AgNPs) to reject negatively-charged ibuprofen and salicylic acid, and the rejection reached 98.1–99.7% and 97.0–99.1%, respectively, owing to the charge repulsion between negatively-charged membrane surface, and ibuprofen/salicylic acid increased steric hindrance by the polymer [22]. However, it has been reported that the rejection of trace organic compounds varies considerably. Yoon et al. reported NF membrane on the dead-end stirred-cell experiments with the PPCP rejection of 30–90% except for naproxen (<10%) and acetaminophen (<25%) [23]. Moreover, in addition to microcontaminants PPCPs, membrane fouling is a major problem that can seriously impede the utilization of membrane technologies. A commonly inorganic fouling source, silica, presences in natural waters in a dissolved state from the weathering of rocks and minerals with concentrations of 1–100 mg·L^−1^ [24]. In Taiwan, another source of silica nanoparticles is the wastewater of wafer polishing in semiconductor factories, which often causes significant membrane fouling for water recycling using membranes. However, according to the authors’ best knowledge, few studies focused on exploring the using in situ modification technology for upgrading membranes that can easily be applied in practical application and can simultaneously reject PPCPs and mitigate colloidal fouling.

Herein, this study in-situ modified a commercial membrane (NF90) by using concentration-polarization-enhanced radical graft polymerization with two types of monomers (SPM and HEMA) to mitigate silica fouling. Membrane permeate flux, fouling reversibility, and membrane surface properties (hydrophilicity and morphology) were analyzed before and after membrane modification. Moreover, the rejection of six commonly detected PPCPs in the aqueous environment with different physicochemical characteristics was evaluated before and after silica fouling, respectively, to confirm the separation performance. Finally, the mechanism of silica fouling was confirmed before and after membrane modification using the modified Hermia model.

## 2. Materials and Methods

### 2.1. Membrane, Chemicals, and Reagents

A commercial thin-film composite (TFC) membrane, NF90, was purchased from Dow FilmTec (Edina, MN, USA) to be modified in this study, which is a polyamide (PA, semiaromatic piperazine-based) NF membrane with the support layers of polysulfone (PSf) and polyester (PET). According to the manufacturer, NF90 has a pure water permeability of 10.56 L·m^−2^·h^−1^·bar^−1^ and high rejection of NaCl and MgSO_4_ (85–95% and >97%, respectively). The physicochemical characteristics of NF90 have been analyzed and reported in previous literature [19,25], including zeta potential of −10.5 mV (at pH 7.1) [26], isoelectric point at pH 4.0 [7], molecular weight cutoff (MWCO) of 200 Da [5,27], average pore radius of 0.34 nm [7], root mean square roughness (Ra) of 142.8 ± 9.6 nm [7], and contact angle of 63.2° [27].

The chemicals for simulating background electrolytes (NaCl and NaHCO_3_), preparing buffer solution (KH_2_PO_4_), and modifying membranes (monomers of SPM and HEMA) and initiators of potassium persulfate (K_2_S_2_O_8_) and potassium metabisulfite (K_2_S_2_O_5_), were all purchased from Sigma Aldrich (New York, NY, USA). SiO_2_ nanoparticles with a purity of 30.4 wt%, particle size of 10–20 nm, and production name of Snowtex were purchased from Nissan Chemical Corp. (Houston, TX, USA) and used to prepare synthetic water with silica fouling potential. Six frequently detected PPCPs were selected to evaluate membrane separation performance, including triclosan (TRI), ibuprofen (IBU), and sulfamethazine (SMZ), carbamazepine (CBZ), sulfadiazine (DIA), and sulfamethoxazole (SMX) (IBU, TRI, and SMZ (purity > 99%) were purchased from Alfa Aesar (Massachusetts, USA), and CBZ, DIA, and SMX (purity > 99%) were purchased from MP Biomedicals (Irvine, CA, USA)). The physicochemical properties of the selected PPCPs are summarized in Table S1 in Supplementary Materials. Based on the acidic dissociation constant (p*K*_a_) and hydrophobicity (log*K_ow_*) at pH 7, the selected PPCPs can be classified as ionic (I)/nonionizable (N) and hydrophobic (HPO)/hydrophilic (HPI), respectively. The detailed information was described in our previous research [28]. Acetonitrile and methanol were used for high-performance liquid chromatography (HPLC) analysis and were purchased from J.T. Baker (NJ, USA).

### 2.2. Experimental Protocols

#### 2.2.1. Filtration Experiments

The filtration system and protocol have been reported in our published studies [16,17,19], and the schematic diagram and the detailed specifications are summarized in Figure S1 and Table S2 in Supplementary Materials. Three identical rectangular cross-flow membrane cells configured in parallel were used to place flat-sheet membrane coupons with each surface area of 137.75 cm^2^. The filtration was performed in a recycle mode and the feed was supplied using a Hydracell diaphragm pump (Wanner Engineering Inc., New York, NY, USA) with the cross-flow velocity of 0.1 m·s^−1^, transmembrane pressure of 690 kPa, and temperature of 25 ± 0.5 °C. The purchased membrane sheet was cut, cleaned, and soaked in deionized (DI) water before use. In each experiment, the membrane coupons were pre-compacted using DI water for 6 h to achieve steady-state permeate flux (J_ps_, L·h^−1^·m^−2^). For salt rejection, 1 g·L^−1^ NaCl was used as the feed solution and operated for 24 h at the same cross-flow velocity, transmembrane pressure, and temperature as previously described. For PPCP rejection by the pristine and modified membranes, 30 L feed solution containing 800 μg·L^−1^ of each PPCP and background electrolytes (20 mM NaCl and 1 mM NaHCO_3_) was used. The rejection of salts and PPCPs is defined as (1−C_p_/C_f_) × 100%, where C_p_ and C_f_ are the concentrations of each target compound in the permeate and feed solutions, respectively. The schematic variation of permeate flux with filtration time is presented in Figure S2.

#### 2.2.2. Membrane Modification

The pre-compacted NF90 coupons were in-situ modified with 10 L feed solution containing a monomer of 0.01–0.05 M SPM or 0.01–0.02 M HEMA and initiators of 0.01 M K_2_S_2_O_8_ and 0.01 M K_2_S_2_O_5_ under the same pressure, cross-flow velocity, and temperature as previously described for 1 h. The schemes of radical graft polymerization using SPM and HEMA for NF90 surface modification were presented in Figure S3a,b, respectively, with explanations given in Text S1 in Supplementary Materials. The dosage of SPM and HEMA were determined considering the polymerization efficiency of the monomers in our preliminary experiments. To remove the residual modification reagents, the filtration system was cleaned thoroughly using DI water at an ambient pressure for 1 h until the pH and conductivity values of the concentrate equaled to those of the DI water. Then, the permeate flux of the modified membranes were measured using DI water at the same pressure, cross-flow velocity, and temperature as previously described and recorded as J_pc_ (L·h^−1^·m^−2^). The flux variation before and after membrane modification was calculated as (J_ps_ – J_pc_)/J_ps_ × 100%. After that, the performance of the modified membrane for salt and PPCP rejection was evaluated according to the protocol described in Section 2.2.1.

#### 2.2.3. Membrane Fouling Experiments

The feed solution containing 1 g·L^−1^ silica with the same background electrolytes was filtrated to form a fouling layer on membrane surface for 24 h under the same pressure, cross-flow velocity and temperature as previously described, to reach a steady-state permeate flux, which was recorded and named J_pss_ as (L·h^−1^·m^−2^). The flux variation after membrane modification and silica fouling was calculated as (J_ps_ – J_pss_)/J_ps_ × 100%. Then, the filtration system was cleaned thoroughly using DI water under ambient pressure for 30 min to remove reversible fouling, and the permeate flux of DI water was measured and named as J_psc_ (L·h^−1^·m^−2^). The flux variation between the initial steady permeate flux (J_ps_) and the permeate flux after removing reversible silica fouling can be calculated as (J_ps_ –J_psc_)/J_ps_ × 100%. Next, the performance of PPCP rejection and the surface characteristics of the fouled modified membranes were measured and analyzed according to the procedures described in Section 2.2.1.

### 2.3. Analytical Methods

#### 2.3.1. Membrane Characterization

The membrane coupons were dried before analyzing their characteristics. The relative atomic concentrations of the elements present in the top surface of the membrane were measured by using X-ray photoelectron spectroscopy (XPS, Thermo Fisher Scientific, England) with Al Kα radiation as the X-ray source (1486.6 eV). Each membrane sample was averaged for five survey scans with electron binding energy (BE) of 0–1200 eV and a resolution of 1 eV. High resolution scans were performed for the element of C, N, and O with the details described in our previous research [16]. The contact angle measurement using the sessile drop method was performed to evaluate the surface hydrophilicity of the membrane coupons using a contact angle meter (Phx mini, Phoenix, Korea) with Milli-Q water as the probe liquid. Each membrane coupon was measured at room temperature (25 ± 1 °C), and Milli-Q water was dropped (2 ± 1 μL) on the membrane surface at more than five different random locations to obtain a convincing data. The structure and morphology of the membranes were evaluated by using a scanning electron microscope (SEM, FEI Quanta 200, Hillsboro, OR, USA) after the membranes were sputtered with a thin layer of gold to enhance conductivity. The 3D images and roughness of the membranes were analyzed and calculated using Image J software (Version 1.53k) of the SEM images.

#### 2.3.2. PPCP Extraction from Membranes

To evaluate the contribution of adsorption on PPCP rejection, the PPCP extraction experiments were performed by manually separating the membrane coupon into the polyamide plus polysulfone layers (PA + PSf) and the polyester (PET) layer and soaking each of the separated membrane layers in 15 mL methanol and oscillated at 25 °C for 24 h. The extracted supernatants were analyzed for PPCP concentrations (μg·m^−2^) using HPLC, and the amount of PPCP concentration (C_A_, μg·L^−1^) per membrane surface area (A, m^2^) can be calculated as (C_A_ × the methanol volume)/(area of extracted membrane).

### 2.4. Confirmation of Fouling Mechanisms

To confirm the silica fouling mechanisms of the pristine and modified membranes, the permeate flux of silica fouling experiments was analyzed using the modified Hermia model, which was developed for cross-flow filtration illustrating four fouling mechanisms [19,29], including complete blocking, incomplete blocking, standard blocking, and gel layer formation. The assumption, equation, and illustration of each fouling mechanism are displayed in Text S2, Table S3, and Figure S4 in the in Supplementary Materials.

## 3. Results and Discussion

### 3.1. Effect of Membrane Modification on Permeate Flux and Surface Characteristics

#### 3.1.1. Before Silica Fouling

**Figure 1:**
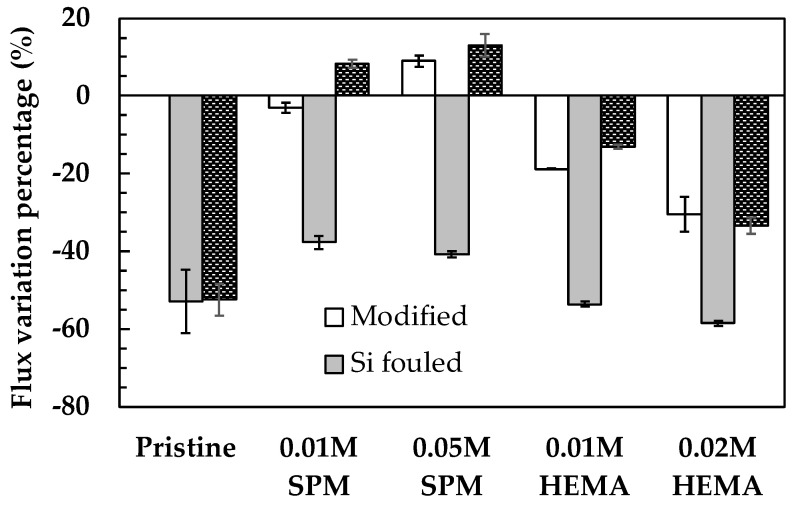
Flux variation of the pristine and modified NF90 after surface modification, silica fouling, and physical cleaning. Error bars represent one standard deviation of triplicate measurements.

The flux variation of the pristine and modified membranes using SPM or HEMA is displayed in Figure 1. The baseline of 0% flux variation was based on the steady-state permeate flux of each pristine membrane coupon after pre-compaction using DI water (J_ps_, described in Section 2.2). As the SPM concentration increased from 0.01 to 0.05 M, the permeate flux of the modified membranes increased from −3.1% to 9.0%. This change could be attributed to the penetration of monomers through the active layer, causing excessive exposure of the polysulfone support to the reactive solution that might hydrolyze and even partly damage membranes pores so as to increase the overall permeability [13]. However, the permeate flux decreased from −18.8% to −30.4% as HEMA concentration increased from 0.01 to 0.02 M. Similar HEMA results were reported for a TFC membrane with the PA layer modified using graft polymerization of methacrylic acid (MA) initiated by plasma for the polymerized time of 60 min [30]. The significant decline in the permeate flux of HEMA modified membranes could be due to the blocking of pores by the graft polymer, resulting in the shrinkage of membrane pore radius and enhancing the concentration polarization of monomer and initiators [13,30].

**Figure 2:**
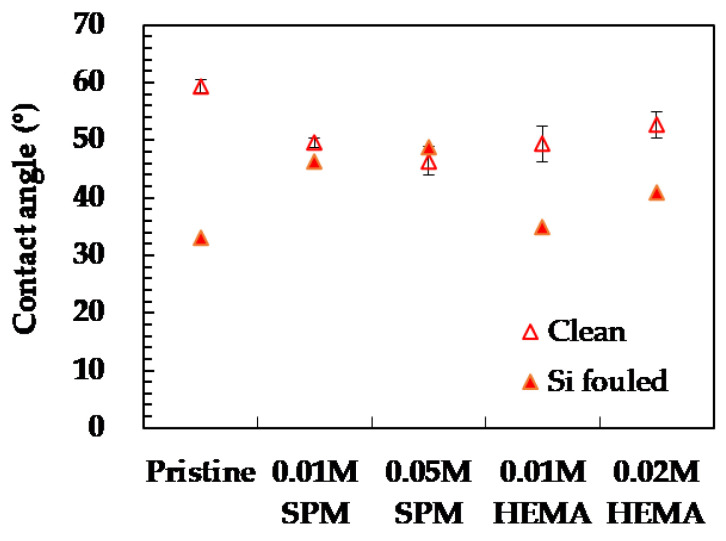
Contact angle of the pristine and modified NF90. Error bars represent one standard deviation of triplicate measurements.

Figure 2 shows the contact angles of the pristine and modified membranes (presented as the hollow symbols). Compared to the pristine membrane (59.4°), the modified membranes both had decreased contact angles (46.3–49.6° using SPM) and (49.7–52.7° using HEMA). The phenomenon indicated that the grafted polymerization using SPM and HEMA could increase the content of membrane carboxylic acid and hydroxyl groups (–COOH and –OH) on the membrane surface, resulting in the enhancement of membrane surface hydrophilicity [31,32]. These hydrophilic groups can form a dense hydration layer via electrostatic interactions, resulting in super water affinity of the modified membranes [33]. Similar results were reported for NF270 membrane modified using SPM and HEMA [13,16].

**Figure 3:**
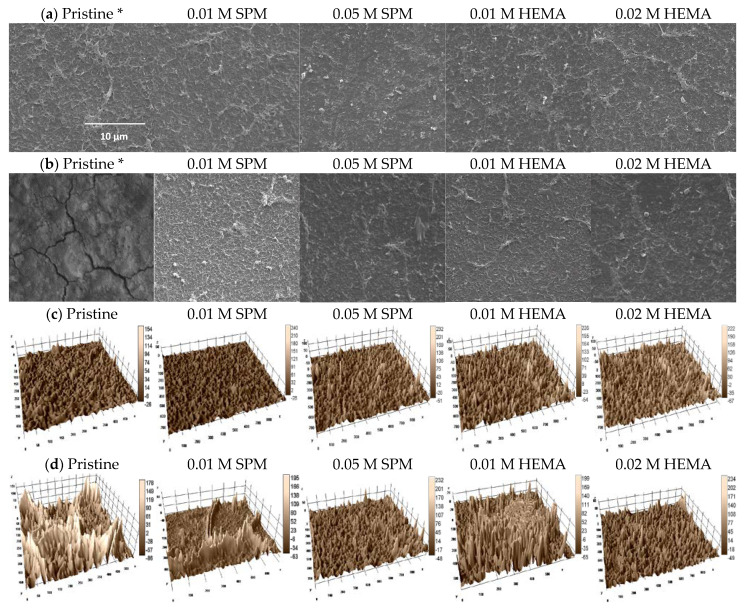
SEM pictures of the pristine and modified NF90 before (**a**) and after (**b**) silica fouling and 3D images of the pristine and modified NF90 before (**c**) and after (**d**) silica fouling. The SEM pictures marked with * were cited from our previous work [25].

Figure 3a shows the SEM pictures of the pristine and modified membranes. The grafted polymers with a typical wrinkled shape of “ridge-and-valley” characteristic structure of PA layer can be clearly seen on the modified membrane surface using SPM and HEMA, especially for the higher monomer concentrations (0.05 M SPM and 0.02 HEMA), in contrast to the relatively smooth surface of the pristine membrane. The observation validated the effectiveness of membrane surface modification, which has also been reported for the modified NF 270 using SPM and HEMA [16]. Figure 3c and Table 1 display the 3D images and roughness data of the pristine and modified membranes, respectively. With the increase of SPM and HEMA concentrations, membrane surface roughness increased for both modified membranes, especially the one using 0.02 M HEMA (Table 1) with more fine crack structures (Figure 3c) on the surface than that using 0.01 M HEMA. This result indicated the coverage and aggregation of grafted higher monomers concentration on the modified membrane surface may lead to the enhancement of membrane roughness. A similar result trend was reported for polysulfone membrane modification by using HEMA [33]. The above statement can be evidenced by the increasing permeate flux of the modified membranes in Figure 1.

**Figure 4:**
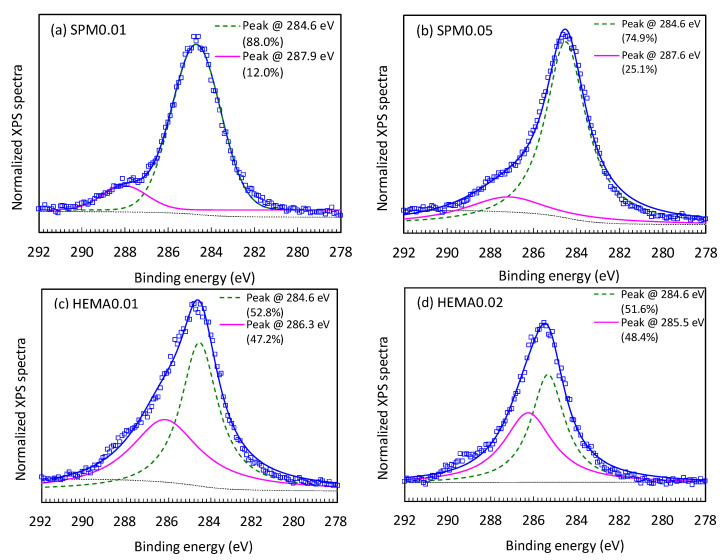
High resolution XPS spectra for modified NF90 using (**a**) 0.01 M SPM, (**b**) 0.05 M SPM, (**c**) 0.01 M HEMA, and (**d**) 0.02 M HEMA.

The element composition on the top 1–5 nm of the pristine and modified membranes surface were measured using XPS, which could further evaluate the effectiveness of membrane modification through this high-resolution technique. The XPS spectra and the surface atomic concentrations of the pristine and modified membranes are presented in Figure 4 and Table 2, respectively, which were normalized against the carbon 1 s peak at 284.6 eV. The carbon, oxygen, and nitrogen are the major elements on the top surface of pristine and modified membranes, which are important key components of PA [34]. Compared to the pristine membrane, all the modified membranes had decreasing carbon content and increasing oxygen and nitrogen contents, leading to an obvious increase in O/C (0.17 and 0.27–0.31) and N/C ratios (0.07 and 0.11–0.13 in Table 2). Figure 4 revealed that the major peak at 284.6 eV was the aromatic and aliphatic carbons, and the secondary peak at 285.5–287.9 eV was the carbons linking to strong electron withdrawing groups such as those in carboxylic and amide groups [35]. The modified membranes using 0.01–0.05 M SPM had higher major peak (74.9–88.0%) than those using 0.01–0.02 M HEMA, indicating more strong electron withdrawing groups in the structures of HEMA modified membranes. Therefore, the results from contact angle (Figure 2), surface morphology and roughness (Figure 3 and Table 1), elemental composition and high resolution XPS analysis (Table 2 and Figure 4) all validated the successful grafting using SPM and HEMA onto NF90.

#### 3.1.2. After Silica Fouling

The flux variation of the pristine and modified membranes after silica fouling (gray-shaded bars) and DI water cleaning (brick-shaded bars) are displayed in Figure 1. A severe flux decline was observed for the pristine membrane after silica fouling (−53.0%), and only 0.5% can be recovered through DI water flushing, which was defined as reversible fouling. Relatively less flux decline was occurred for all the SPM modified membranes, and they exhibited much higher reversible fouling percentages than the pristine membrane did. On the other hand, the flux performance of the modified membranes using SPM was better than those modified using HEMA. HEMA modified membranes had a higher flux decline (−53.6% to −58.5%) after silica fouling. However, compared to the pristine membrane, HEMA modified membranes exhibited significantly higher permeate flux recovering after DI cleaning (−13.1% to −33.4%). At higher monomer concentrations, 0.05 M SPM or 0.02 M HEMA more severe permeate flux decline was observed, which could be due to more pore blocking by the grafted polymers [37], leading to the decrease of effective membrane pore radius [38] so as for more deposition of silica particles on membrane surfaces. Overall, the modified membranes all exhibited superior reversible fouling compared to the pristine one, implying that the in-situ membrane modification technique has high potential for mitigating silica fouling. Figure 2 shows the contact angles of the pristine and modified membranes after silica fouling (presented as the solid symbols). The contact angles of all the silica fouled membranes were lower than the pristine membrane, which could be attributed to the deposition of highly hydrophilic silica nano-particles that have abundant silanol groups (–Si–OH) with strong affinity to water [38,39]. Moreover, the contact angle of silica fouled modified membranes slightly increased with increasing monomer concentration (from 46.3° to 48.9° for SPM and from 35.0° to 41.0° for HEMA), implying less silica fouling on the membrane surface, which correlate well to the high reversible flux percentage displayed in Figure 1.

Figure 3b present the SEM images of the surface morphology of the pristine and modified membranes after silica fouling, respectively. There was a thick and dense silica cake with cracks on the surface of the silica-fouled pristine membrane (Figure 3b). However, there were significantly less silica deposition on the surface of modified membranes compared to the pristine one, and the characteristic “ridge-and-valley” structures of the PA layer on the surface of the modified membranes were obvious, which also validated the effectiveness of in-situ membrane modification for mitigating silica fouling. Figure 3d and Table 2 present the 3D images and the surface roughness of the pristine and modified membranes, respectively, before and after silica fouling. With the increase of SPM and HEMA concentrations, a slight decrease of surface roughness was observed for both modified membranes after silica fouling, indicating less fine crack structures (Figure 3d) compared to the pristine membrane surface. The pristine membrane surface may lead to the enhancement of membrane roughness because of uncovering the grafted monomers on the membrane surface. Overall, membranes modified using a lower monomer concentration (0.01 M SPM or 0.01 M HEMA) exhibited less flux decline after surface modification and high percentage of reversible fouling (Figure 1), which was more cost-effective in practical application.

### 3.2. Effect of Membrane Modification on Salt Rejection

Figure S5 displays the results of salt rejection by the pristine and modified membranes. All the modified membranes achieved a considerably higher NaCl rejection (86.2–100%) compared with the pristine membrane (80.4%), which could be attributed to the Donnan exclusion effect due to the negatively charged polyamide layer on the membrane surface, contributing to the retention of the tiny and mono-charged NaCl [40,41]. The high NaCl rejection indicated that NF90 is a rather tight NF membrane compared to other NF membranes (such as NF270 with NaCl rejection of approximately 30% [19]). NF90 modified using a higher monomer SPM concentration of 0.05 M had slightly decreasing salt rejection (Figure S5), which could be due to the penetration of monomer into the supporting layer that could hydrolyze and even partially damage membrane pores, resulting in the increase of permeability (Figure 1). Similar phenomena were observed for NF270 modified using 0.05 M SPM in our previous study [16]. On the other hand, the functional groups of SPM and HEMA are different, which can cause different effects in the same concentrations of SPM and HEMA on the membrane modification. Therefore, this membrane modification technique should be implemented with low SPM or HEMA concentrations to upgrade membrane performance cost-effectively.

### 3.3. Effect of Membrane Modification on PPCP Rejection and Adsorption

#### 3.3.1. Before Silica Fouling

**Figure 5:**
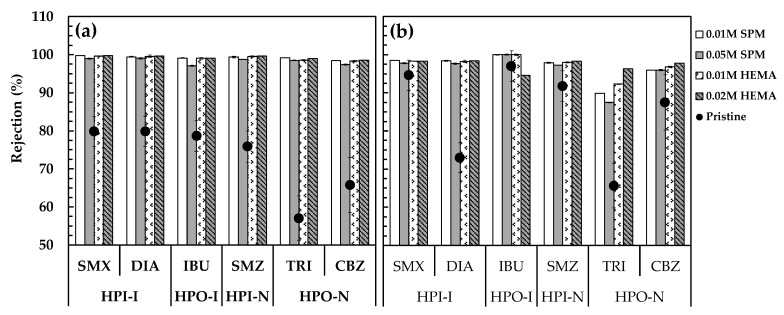
The removal of PPCPs by the pristine and modified NF90 before (**a**) and after (**b**) silica fouling. Error bars represent one standard deviation of triplicate measurements. SMX: sulfamethoxazole, DIA: sulfadiazine, IBU: ibuprofen, SMZ: sulfamethazine, TRI: triclosan, CBZ: carbamazepine. The data of the pristine membrane were summarized from our previous work [19,42].

**Figure 6:**
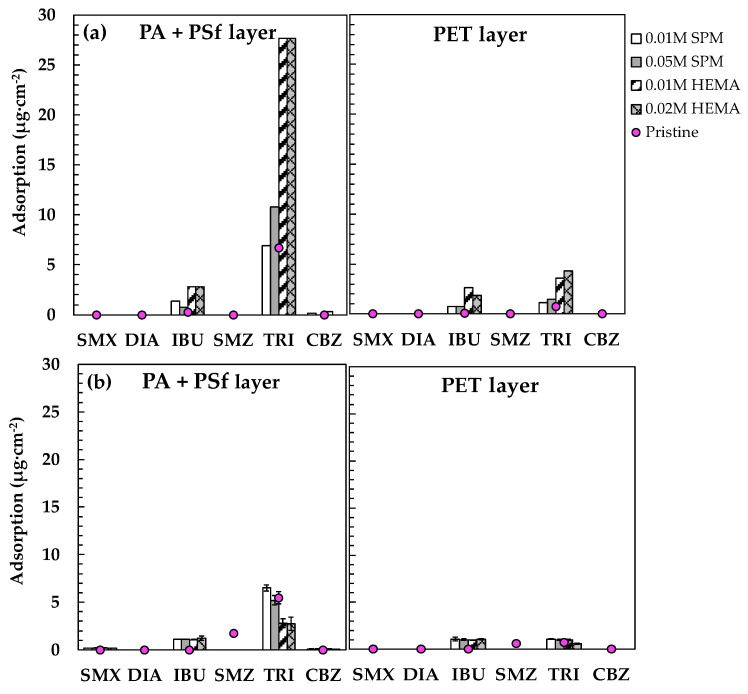
Adsorption of IBU and TRI extracted from PA + PSf and PET layers of the pristine and modified NF90 before (**a**) and after (**b**) silica fouling. Error bars represent one standard deviation of triplicate measurements. SMX: sulfamethoxazole, DIA: sulfadiazine, IBU: ibuprofen, SMZ: sulfamethazine, TRI: triclosan, CBZ: carbamazepine. The data of pristine membrane were summarized from our previous work [19,42].

Figure 5a displays the removal of PPCPs by the pristine and modified NF90 before silica fouling. The pristine membrane exhibited obviously higher rejection of the hydrophilic ionized SMX, DIA, and SMZ, and hydrophobic ionized IBU (75.9% to 79.9%, round dots in Figure 5a) because of the synergistic effect of size exclusion and electrostatic repulsion for the ionized PPCPs [17]. For the highly hydrophobic non-ionized (HPO-N) TRI and CBZ, their rejection was relatively low (57.0% and 65.8%, in Figure 5a) because of the only rejection mechanism of steric hindrance without electrostatic repulsion [20]. The significantly low rejection of TRI was caused by its adsorption higher on membrane surface (Figure 6a) and penetration through membrane pores (Figure 6b). On the contrary, the SPM and HEMA modified membranes exhibited remarkably increasing rejection of the six PPCPs (97.1–99.8%, bars in Figure 5a), indicating that the grafted polymer may form both an extra steric barrier layer, enhancing steric hindrance effect and also contribute to electrostatic repulsion effect for the removal of PPCPs [1]. According to our previous research, NF270 modified using SPM and HEMA significantly increased the negatively zeta potential on membrane surface [16] (Figure S6a), which is similar with NF90 because the active layer of both membranes is polyamide. Therefore, it is rationed to assume that increasing negative surface charge on the surface of NF90 will occur, leading to enhancing electrostatic repulsion between the modified NF90 and ionized PPCPs, as that has confirmed for the modified MF270 using the same monomers. Although the adsorption amount of the highly hydrophobic IBU and TRI both increased on the surface of modified membranes (especially using HEMA), they can be well retained by the modified membrane without penetration.

#### 3.3.2. After Silica Fouling

Figure 5b displays the removal of PPCPs by the pristine and modified NF90 after silica fouling. Compared with the unfouled pristine membrane (Figure 5a), most of the PPCP rejection by the silica-fouled pristine membrane considerably increased by 8.6–21.7% except for DIA (declined by 6.9%), implying that the silica fouling layer may serve as additional steric hindrance to retain the PPCPs [16,43], and there could be strong affinity between the hydrophilic silica and DIA so as to facilitate its penetration through membrane [44]. The affinity between silica and DIA was also observed in other studies [45], which may be attributed to its higher hydrophilicity (log *K*_ow_ = 0.21) as that of silica. On the other hand, the PPCP rejection of the silica-fouled modified membranes all remained high (94.5–100.0%). Figure 6b displays the adsorption of PPCPs on the silica-fouled pristine and modified membranes. The highly hydrophobic TRI and IBU were the major adsorbed PPCPs on membrane surface, but the adsorption amount significantly decreased compared to the unfouled pristine membranes (Figure 6a). The results imply that the adsorption of both IBU and TRI should happen on the grafted polymers and PA, and the presence of highly hydrophilic silica nanoparticles inhibited their access to membrane surface, leading to considerably decreasing adsorption. On the other hand, the adsorption of hydrophilic SMZ happened on the silica-fouled membranes, implying that the adsorption happened on silica fouling layer instead of membrane surface. Similar phenomena were observed for the silica-fouled NF90, NF270, and XLE membranes at pH 3–10 in our previous report [19].

### 3.4. Confirmation of the Silica Fouling Mechanisms

**Figure 7:**
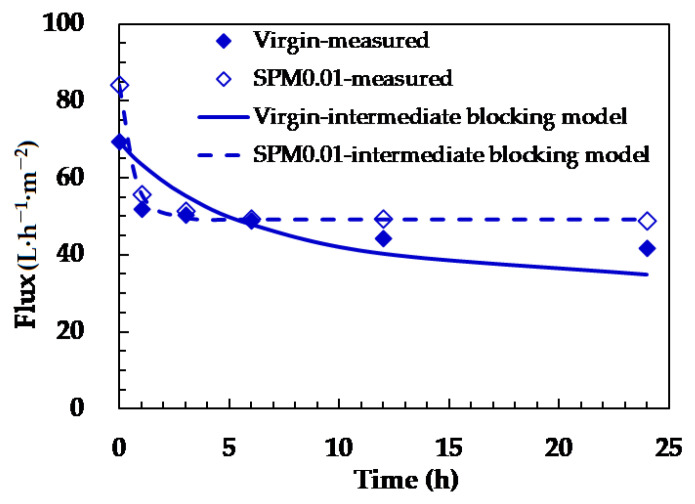
Experimental and model predicted flux as a function of time for the pristine and 0.01 M SPM modified NF90 during silica fouling. (L·h^−1^·m^−2^).

Table 3 summarizes the fitted Hermia model constant related to the nature of fouling (K values) and goodness of data fitting (R^2^) of the pristine and modified membranes. The silica fouling mechanism of the pristine (R^2^ = 0.91) and modified (R^2^ = 0.94–1.00) membranes was all determined to be incomplete blocking with R^2^ approaching 1.00. The incomplete blocking of membrane pores can be owing to the ridge-and-valley structure of membrane surface so that the round silica nanoparticles cannot easily block all the pores. Compared to the pristine membrane, the modified membranes exhibited the lower K values (Table 3) correlate well to less flux decline after 3 h (from 49.8 to 49.3·h^−1^·m^−2^ in 3–24 h, Figure 7). The best fitted results of the membrane modified using SPM 0.01 M (R^2^ = 1.00) along with those of the pristine one are displayed in Figure 7. The flux of the pristine membrane continuously declined from 69.4 to 35.0 L·h^−1^·m^−2^ in 24 h, while the flux of the modified membrane rapidly decreased in the first hour and remained stable in the following (from 84.1 to 49.3 L·h^−1^·m^−2^ in 24 h). These results indicated that silica particles partially blocked the pores of the pristine and modified membranes, shrinking the passage to water molecules and causing flux decline.

## 4. Conclusions

This study in-situ modified NF90 using two different monomers of SPM and HEMA to mitigate to silica fouling and enhance the PPCP rejection. Results showed that all the modified membranes had rougher membrane morphology, superior hydrophilicity, and considerably less silica fouling with higher reversible fouling compared to the pristine membrane. Especially, the modified membranes using low monomer concentration (0.01 M SPM and 0.01 M HEMA) exhibited less flux decline and higher rejection of both NaCl and PPCPs compared to those modified using higher monomer concentrations. The fouling mechanism was confirmed to be the intermediate blocking of membrane pores with considerably lower K values of the modified membranes than that of the pristine membrane. Therefore, the in-situ modification of NF90 proved to be effective for mitigating silica fouling and improving NaCl and PPCP rejection, especially at low monomer concentrations, which can be more cost-effective in practical application.

## Figures and Tables

**Table 1:** Surface characteristics of the pristine and modified NF90 before and after silica fouling.

Membrane	Silica Fouling	Surface Morphology	Rq ^a^ (μm)	Ra ^b^ (μm)
Pristine	No	Smooth valley	8.0	6.0
0.01 M SPM	No	Ridge valley	8.3	6.1
0.05 M SPM	No	Ridge valley	18.2	12.9
0.01 M HEMA	No	Ridge valley	20.7	15.0
0.02 M HEMA	No	Ridg valley	22.9	17.0
Pristine	Yes	Ridge valley	40.8	33.0
0.01 M SPM	Yes	Ridge valley	18.0	12.1
0.05 M SPM	Yes	Ridge valley	16.5	11.8
0.01 M HEMA	Yes	Ridge valley	19.1	13.8
0.02 M HEMA	Yes	Ridge valley	16.6	11.6

^a^ Root mean square deviation of surface roughness. ^b^ Arithmetical mean deviation of surface roughness.

**Table 2:** Surface atomic concentrations of the pristine and modified NF90.

Membrane		C (%)	O (%)	N (%)	O/C	N/C	O/N
Pristine ^a^		75.99	13.22	5.52	0.17	0.07	2.39
Modified	0.01 M SPM	71.22	19.65	9.14	0.28	0.13	2.15
	0.05 M SPM	71.72	19.64	8.63	0.27	0.12	2.28
	0.01 M HEMA	70.32	21.89	7.79	0.31	0.11	2.81
	0.02 M HEMA	70.38	21.06	8.56	0.30	0.12	2.46

^a^ Data from Suo and Ren (2021) [36].

**Table 3:** K values and the goodness of fit (R^2^) for silica-fouled pristine and modified NF90 determined using the modified Hermia model.

Membrane		K_c_^b^ (1·m^−1^)	R^2^	K_i_^c^ (1·m^−1^)	R^2^	K_s_^d^ (10^−3^·s^−0.5^·m^−0.5^)	R^2^	K_gl_^e^ (10^−5^·s·^m−2^)	R^2^
Pristine ^a^		1.95	0.80	13.30	0.91	6.27	0.63	3.36	0.82
Modified	SPM0.01	0.02	1.00	0.02	1.00	0.01	0.41	NA	NA
	SPM0.05	0.01	0.90	0.02	0.94	0.01	0.63	NA	NA
	HEMA0.01	0.02	0.94	0.03	0.96	0.01	0.53	NA	NA
	HEMA0.02	0.02	0.98	0.03	0.99	0.01	0.57	NA	NA

^a^ Data from Suo and Ren (2021) [36]. ^b^ Fitting parameter for completely blocking. ^c^ Fitting parameter for intermediate blocking. ^d^ Fitting parameter for standard blocking. ^e^ Fitting parameter for gel layer formation.

## Data Availability

This study did not report any data in public datasets analyzed or generated.

## Supplementary Materials

The following are available online at https://www.mdpi.com/article/10.3390/membranes11110904/s1, Table S1: Physicochemical properties of the selected PPCPs in this study, Table S2: The parameters and the detailed specifications for theparallel rectangular cross-flow filtration system, Table S3: The modified Hermia model for the simulation of silica fouling, Figure S1: The schematic diagram of the cross-flow filtration system, Figure S2: The schematic variation of permeate flux with filtration time, Figure S3: Schemes of radical graft polymerization using (a) SPM and (b) HEMA for NF90 surface modification, Figure S4: Illustration of the fouling mechanisms by the models: (a) complete blocking, (b) incomplete blocking, (c) standard blocking, and (d) gel layer formation, Figure S5: Salt rejection by the pristine and modified NF90, Figure S6: Surface zeta potential of the pristine and modified (a) NF90 and (b) NF270, Text S1: The mechanism of the radical graft polymerization for membrane surface modification, Text S2: The modified Hermia model.
