# Supplementary material for: Mitigating Silica Fouling and Improving PPCP Removal by Modified NF90 Using In Situ Radical Graft Polymerization
Source: Membranes (Basel). 2021 Nov 22;11(11):904. doi: 10.3390/membranes11110904 (PMC8618697; doi:10.3390/membranes11110904)
Supplement: Supplementary file 1 [file membranes-11-00904-s001.zip › membranes-1468539-supplementary.pdf]

membranes

Supporting Information

Mitigating Silica Fouling and Improving PPCP Removal by Modified NF90 Using in Situ Radical Graft Polymerization

Yi-Li Lin *, Nai-Yun Zheng, Hao-Yu Gan, An-Xian Chang, Huai-Xuan Luo and Yao-Jie Mao

Department of Safety, Health and Environmental Engineering, National Kaohsiung University of Science and Technology, Kaohsiung 824, Taiwan; naiyun@nkust.edu.tw (N.-Y.Z.); u0313054@nkust.edu.tw (H.-Y.G.); afsd124afsd@gmail.com (A.-X.C.); u0313074@nkust.edu.tw (H.-X.L.); u0313006@nkust.edu.tw (Y.-J.M.)
* Correspondence: yililin@nkust.edu.tw

Table S1. Physicochemical properties of the selected PPCPs in this study.

Name (Abbreviations)	Structure	Molecular formula	Molecular weight (g/mol)	Diffusion coefficient (10^{-10} m²/s)[a]	Stokes radius (nm)[a]	p*K*a[b]	log*K*ow[c]	Classification[d]
Carbamazepine (CBZ)	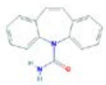	$C_{15}H_{12}N_2O$	236.3	5.81	0.422	13.9	2.45	HPO-N
Ibuprofen (IBU)	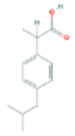	$C_{13}H_{18}O_2$	206.3	5.95	0.412	4.3	3.14	HPO-I
Sulfadiazine (DIA)	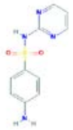	$C_{10}H_{10}N_4O_2S$	250.3	6.11	0.401	6.4	0.21	HPI-I
Sulfamethoxazole (SMX)	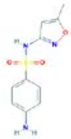	$C_{10}H_{11}N_3O_3S$	253.3	6.08	0.403	5.7	0.86	HPI-I
Sulfamethazine (SMZ)	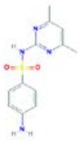	$C_{12}H_{14}N_4O_2S$	278.3	5.58	0.439	7.6	1.62	HPI-N
Triclosan (TRI)	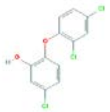	$C_{12}H_7Cl_3O_2$	289.5	5.91	0.415	8.0	4.86	HPO-N

[a] Calculated from the method proposed by Wilke and Chang [1]. [b] ADME/Tox Web Software. [c] Calculated using ChemOffice 2010. [d] HPI: hydrophilic (log K_{ow} ≦ 2), HPO: hydrophobic (log K_{ow} > 2), I: ionic (pK_a ≦ 7), N: non-ionic (pK_a > 7).

Table S2. The parameters and the detailed specifications for theparallel rectangular cross-flow filtration system are given in the following:.

Apparatus or instrument	Specification
Water bath	Model：Water Bath D-606, DENG YNG, Taiwan.<br>Control the feed water temperature at 25 ± 0.5 °C.
Feedwater reservoir	30 L, polyethylene (PE), Taiwan.
Cross-flow filtration module	A self-designed, cross-flow mode filtration apparatus with a flat-sheet membrane cell.<br>All parts of the experimental apparatus were made of stainless steel.<br>The effective membrane area：137.75 cm^2.
High pressure pump	Hydracell diaphragm pump (Wanner Engineering Inc., USA)
Pressure gauge	Operating condition：6.9 kg/cm^2 (100 psi).
Flow meter	Operating condition: 1.14 L/min.
Pressure regulation valve	1/4″ Stainless steel 316.

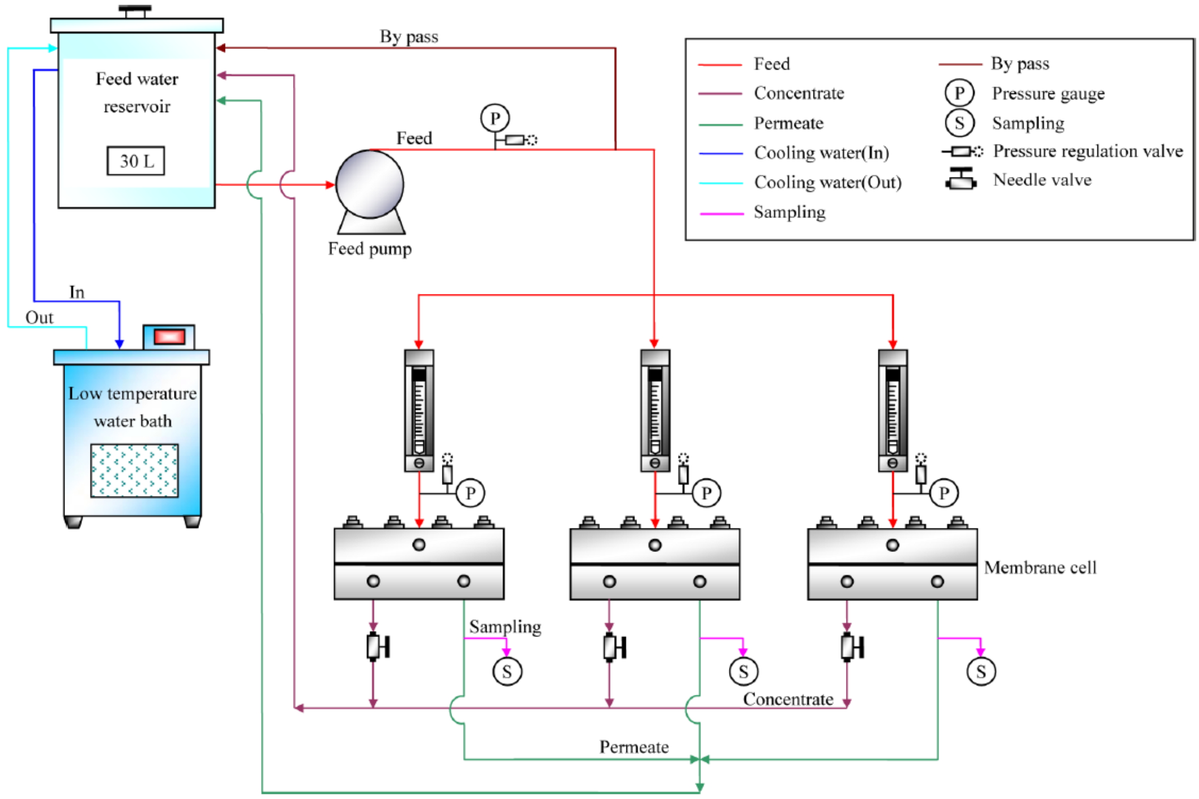

Figure S1. The schematic diagram of the cross-flow filtration system [2].

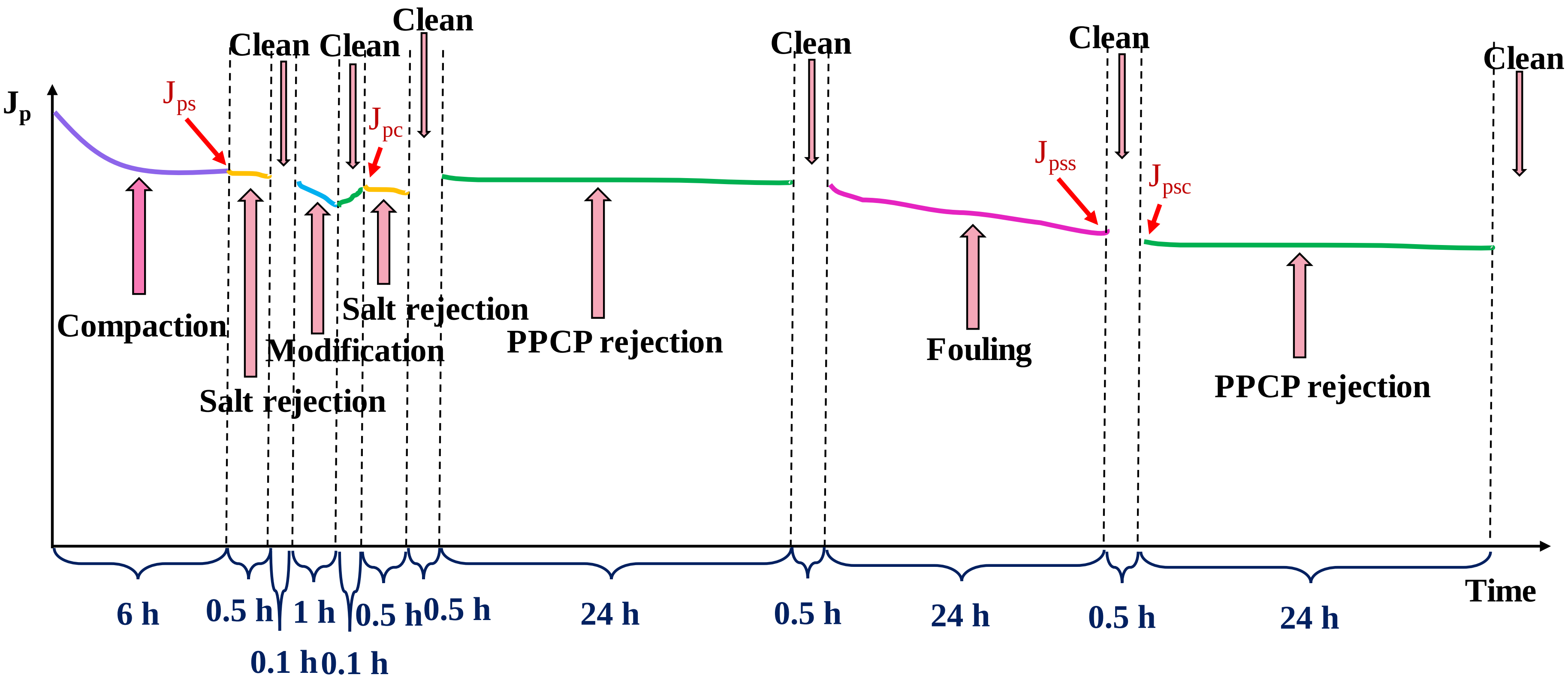

Figure S2. The schematic variation of permeate flux with filtration time.

Text S1: The mechanism of the radical graft polymerization for membrane surface modification

The radical graft polymerization for membrane surface modification is generally used a radical monomer such as 3-sulfopropyl methacrylate potassium salt (SPM) and 2-hydroxyethyl methacrylate (HEMA). During radical graft polymerization, monomer is used to the copolymerization for membrane surface modification with different functional groups (such as single –OH, $–NR_2$, and –COOH) and initiator is used to initiate the reactioco of monomer on the membrnae surface. On the other hand, graft polymerization is initiated by radicals on the substrate formed during graft process that can produce and form the radicals and free hydroxyl radicals on the substrate in addition initiator. An initiation reaction by a hydroxyl radical or a propagation reaction with a growing polymer chain and crosslinking reaction with an adjacent growing grafted chain will make the growing polymer chains connected to the substrate and thus enhance separation performance of trace contaminant by the modified membranes.

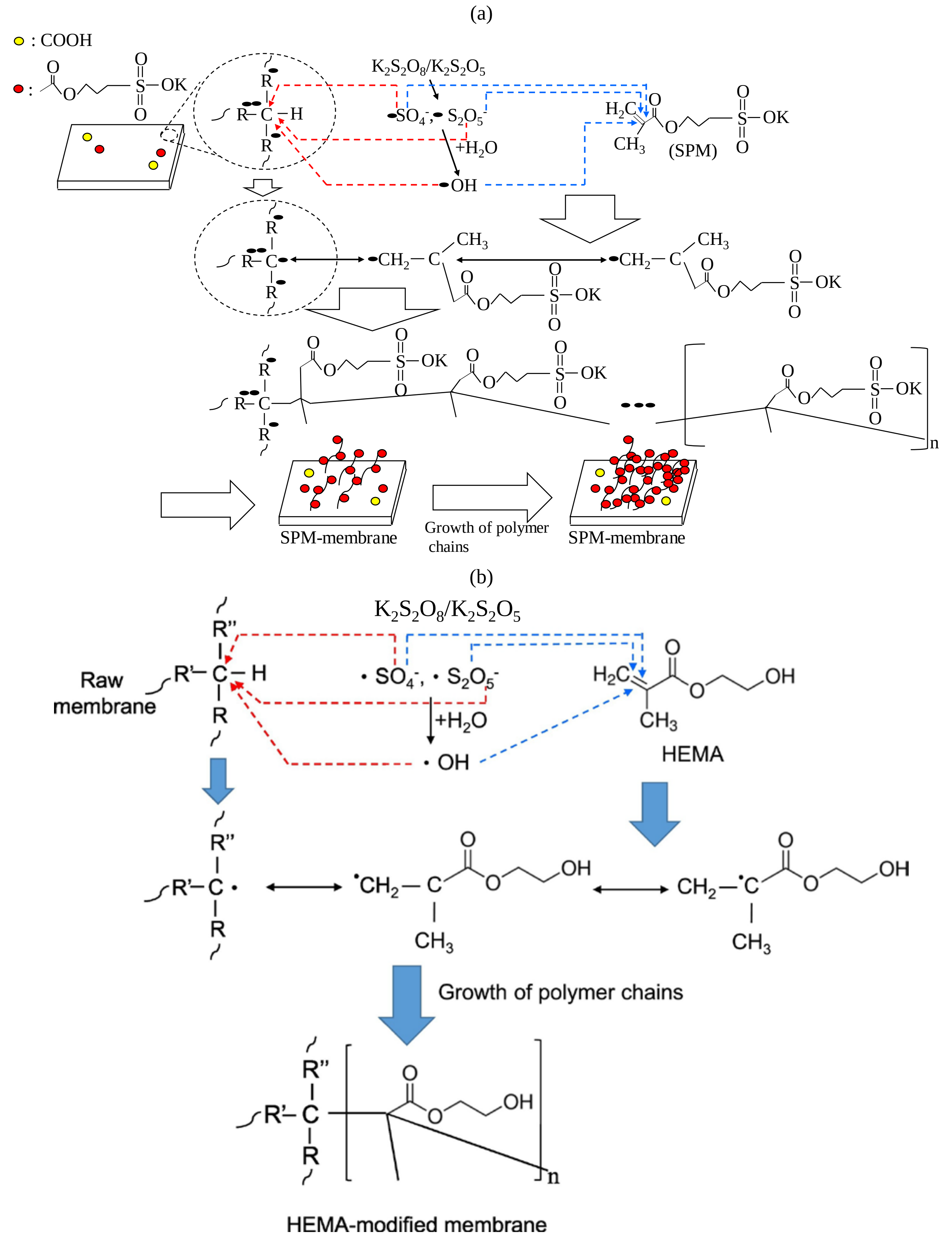

Figure S3. Schemes of radical graft polymerization using (a) SPM and (b) HEMA for NF90 surface modification, which are edited from our previous work [3, 4].

Text S2: The modified Hermia model

The assumptions of the fouling mechanisms were briefly given as follows: (1) complete blocking (Eq. (1)): each particle blocks a membrane pore without superposition, and

decreases the effective membrane area, (2) incomplete blocking (Eq. (2)): also called intermediate blocking, is some particles partially block membrane pores, but other particles can settle over the blocking particles, (3) standard blocking (Eq. (3)): particles smaller than the membrane pores settle on the inner pore wall due to adsorption and cause the pore to constrict, and (4) gel layer formation (Eq. (4)): also called cake-layer fouling, is clogging of the membrane pores by the deposition of particles on the membrane surface and form a cake. All the fouling mechanisms may lead to a decrease in filtrate volume [5].

Table S3. The modified Hermia model for the simulation of silica fouling.

Fouling Mechanism	Equation	
Completely blocking	$J_p = J_{pss} + (J_{pc} - J_{pss})e^{-K_c J_0 t}$	(1)
Intermediate blocking	$J_p = \frac{J_{pc} J_{pss} e^{K_i J_{pss} t}}{J_{pss} + J_{pc}(e^{K_i J_{pss} t} - 1)}$	(2)
Standard blocking	$J_p = \frac{4J_{pc}}{(2 + J_{pc}^{1/2} K_s t)^2}$	(3)
Gel layer formation	$t = \frac{1}{K_{gl} J_{pss}^2}\left[\ln\left(\frac{J_p}{J_{pc}} \cdot \frac{J_{pc} - J_{pss}}{J_p - J_{pss}}\right) - J_{pss}(\frac{1}{J_p} - \frac{1}{j_{PC}})\right]$	(4)

J_{pc}: permeate flux of clean modified membrane (m/s). J_{pss}: steady-state permeate flux of modified membrane after silica fouling for 24 h (m/s). J_p: the simulated flux of fouled membrane (m/s). K_c: the blocked membrane surface per unit of permeated volume through membrane and initial membrane porosity (1/m). K_i: the blocked membrane surface per unit of permeated volume through membrane and initial membrane porosity (1/m). K_s: the volume of retained particle per unit of permeated volume through membrane, membrane thickness and initial membrane porosity ($1/s^{0.5}m^{0.5}$). K_{gl}: the ratio of gel layer resistance and intrinsic membrane resistance times the gel layer mass per unit of permeated volume (s/m^2). t: filtration time (s).

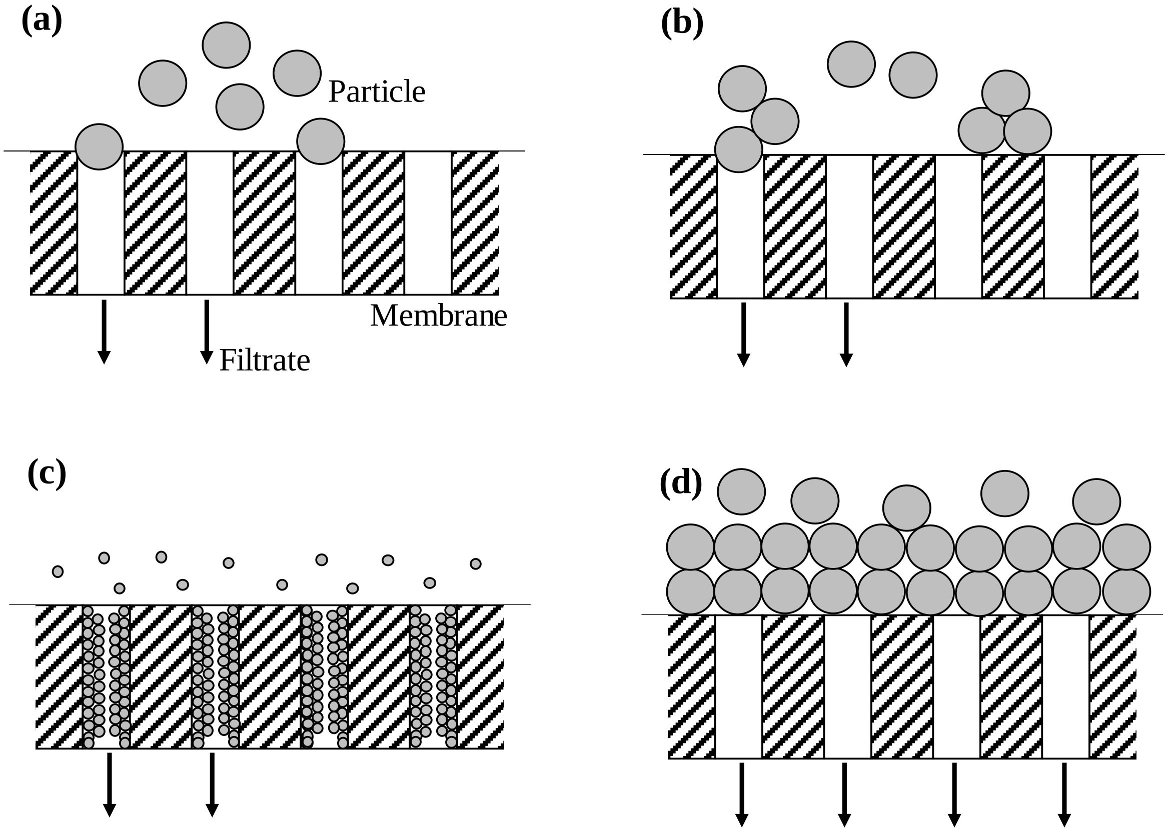

Figure S4. Illustration of the fouling mechanisms by the models: (**a**) complete blocking, (**b**) incomplete blocking, (**c**) standard blocking, and (**d**) gel layer formation.

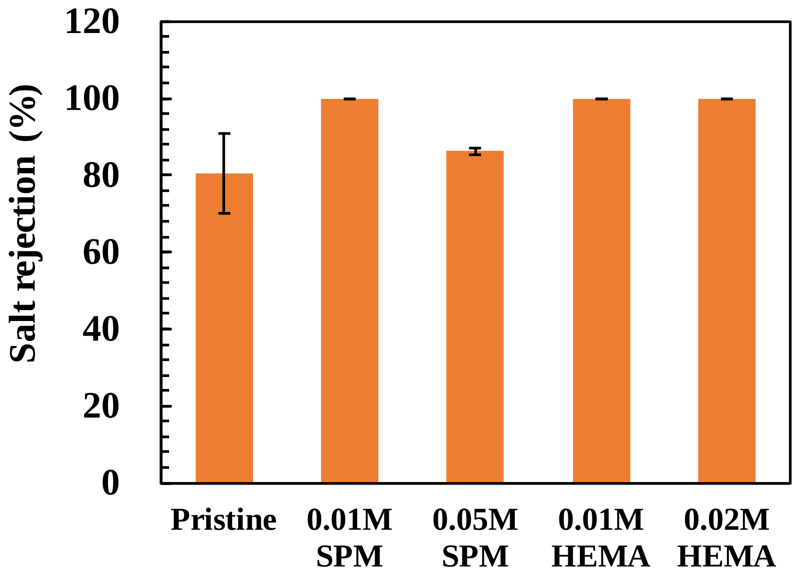

Figure S5. Salt rejection by the pristine and modified NF90 [6]. Error bars represent one standard deviation of triplicate measurements.

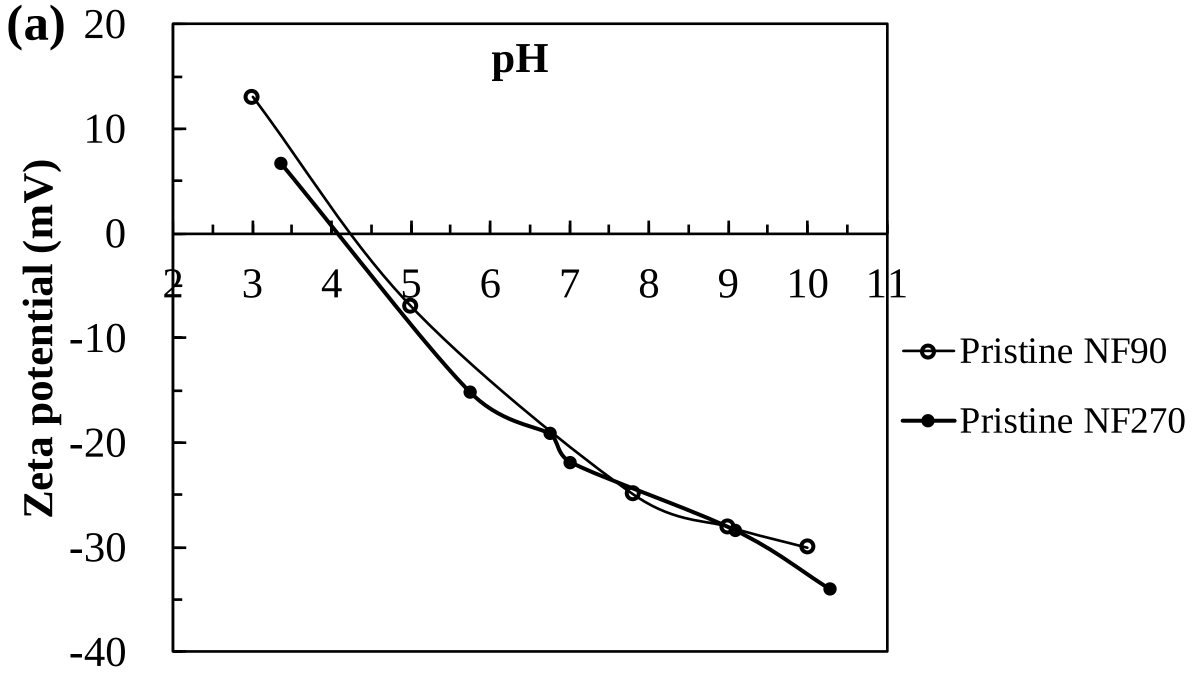

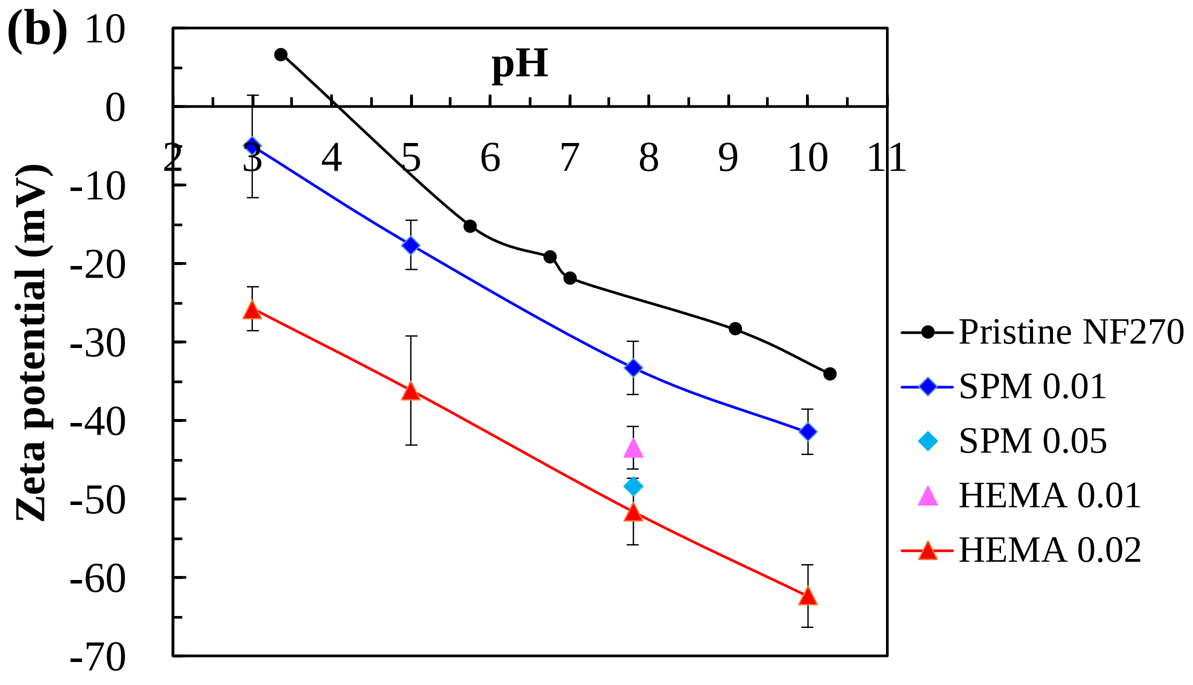

Figure S6. Surface zeta potential of the pristine and modified (a) NF90 [7] and (b) NF270 [8]. Error bars represent one standard deviation of six measurements.

References

[1] C. Wilke, P. Chang, Correlation of diffusion coefficients in dilute solutions, AIChE Journal, 1 (1955) 264-270.

[2] Y.L. Lin, C.H. Lee, Elucidating the Rejection Mechanisms of PPCPs by Nanofiltration and Reverse Osmosis Membranes, Industrial and Engineering Chemistry Research, 53 (2014) 6798-6806.

[3] Y.L. Lin, C.C. Tsai, N.Y. Zheng, Improving the organic and biological fouling resistance and removal of pharmaceutical and personal care products through nanofiltration by using in situ radical graft polymerization, Science of The Total Environment, 635 (2018) 543-550.

[4] J.J. Cai, In situ modification of NF membranes for enhancing anti fouling ability and PPCP rejection (Master's thesis), Department of Safety, Health and Environmental Engineering, National Kaohsiung Firist University of Science and Technology, Kaohsiung, Taiwan, (2016).

[5] I.A. Khan, Y.S. Lee, J.O. Kim, A comparison of variations in blocking mechanisms of membrane-fouling models for estimating flux during water treatment, Chemosphere, 259 (2020) 127328.

[6] Y.L. Lin, J.Z. Tsai, C.H. Hung, Using in situ modification to enhance organic fouling resistance and rejection of pharmaceutical and personal care products in a thin-film composite nanofiltration membrane, Environmental Science and Pollution Research, 26 (2019) 34073-34084.

[7] Y.-L. Lin, Effects of organic, biological and colloidal fouling on the removal of pharmaceuticals and personal care products by nanofiltration and reverse osmosis membranes, Journal of Membrane Science, 542 (2017) 342-351.

[8] Y.-L. Lin, In situ concentration-polarization-enhanced radical graft polymerization of NF270 for mitigating silica fouling and improving pharmaceutical and personal care product rejection, Journal of Membrane Science, 552 (2018) 387-395.
